# Ratio Indexes Based on Spectral Electroencephalographic Brainwaves for Assessment of Mental Involvement: A Systematic Review

**DOI:** 10.3390/s23135968

**Published:** 2023-06-27

**Authors:** Ilaria Marcantoni, Raffaella Assogna, Giulia Del Borrello, Marina Di Stefano, Martina Morano, Sofia Romagnoli, Chiara Leoni, Giulia Bruschi, Agnese Sbrollini, Micaela Morettini, Laura Burattini

**Affiliations:** Department of Information Engineering, Engineering Faculty, Università Politecnica delle Marche, 60131 Ancona, Italy; i.marcantoni@staff.univpm.it (I.M.); S1104460@studenti.univpm.it (R.A.); S1104933@studenti.univpm.it (M.D.S.); S1107637@studenti.univpm.it (M.M.); s.romagnoli@pm.univpm.it (S.R.); chiara.leoni.940@gmail.com (C.L.); g.bruschi@pm.univpm.it (G.B.); a.sbrollini@staff.univpm.it (A.S.)

**Keywords:** electroencephalogram, engagement index, vigilance, mental involvement, brainwaves, brain rhythms, ratio index

## Abstract

Background: This review systematically examined the scientific literature about electroencephalogram-derived ratio indexes used to assess human mental involvement, in order to deduce what they are, how they are defined and used, and what their best fields of application are. (2) Methods: The review was carried out according to the Preferred Reporting Items for Systematic Review and Meta-Analyses (PRISMA) guidelines. (3) Results: From the search query, 82 documents resulted. The majority (82%) were classified as related to mental strain, while 12% were classified as related to sensory and emotion aspects, and 6% to movement. The electroencephalographic electrode montage used was low-density in 13%, high-density in 6% and very-low-density in 81% of documents. The most used electrode positions for computation of involvement indexes were in the frontal and prefrontal cortex. Overall, 37 different formulations of involvement indexes were found. None of them could be directly related to a specific field of application. (4) Conclusions: Standardization in the definition of these indexes is missing, both in the considered frequency bands and in the exploited electrodes. Future research may focus on the development of indexes with a unique definition to monitor and characterize mental involvement.

## 1. Introduction

During daily life, we perform a series of physical and cognitive activities that are managed and controlled by the brain. Indeed, the brain controls emotions, thought, memory, touch, sensory responses, motor skills, movement management, vision processing, language processing, breathing, temperature, hunger and, in general, every process that regulates our body. In particular, while performing continuous long-lasting (or even repetitive) activities, subjects’ brains are involved in functions that require sustained attention and, more generally, mental involvement.

Mental involvement can be defined as a brain state of focused vigilance, commitment, and active involvement, which is maintained over time while focusing on a task [1]. In this state there is a kind of alienation of an individual’s sphere of awareness and attention from the rest of the world. While mentally engaged, the subject is able to maintain mental alertness and participation on the specific task [2]. In this, there is not a single instantaneous event (of a different possible nature, e.g., acoustic, visual, etc.) to which the subject is expected to respond within a certain time interval to demonstrate the ability of mental readiness, but rather a series of continuous stimuli that require a high and continuous level of alertness. Furthermore, from a comprehensive perspective, mental involvement includes behavioral and emotional involvement, not only cognitive involvement [1].

In several fields of application, it is useful to assess the level of mental involvement. In recent years, assessment of mental involvement has gained popularity in working environments and other fields since it was proved that it provides a measure of stress that, if excessive, could jeopardize the mental well-being status of individuals and even lead to mistakes and injuries with possible consequences for the individual and others [3,4]. Besides working environments, mental involvement is also an important aspect in virtual reality applications. Virtual reality is entering many areas of life, not only recreational and game development but also the medical field. In virtual reality different levels of immersivity (i.e., immersion intensity) can be defined: from a low-immersive virtual reality experience to a high-immersive virtual reality experience, which are differentiated essentially based on the sensory equipment used to experience the virtual environment. In this context, assessment of the immersivity level could be useful and we can affirm that the concept of immersivity is closely connected with that of mental involvement. The fields of application of mental involvement assessment could be many others. They can range from the commercial field to evaluate the effectiveness of an advertising medium to the medical field for the evaluation of cognitive abilities and attention deficit disorders. In this context, the possibility of assessing mental involvement is of relevance and should be systematically addressed.

Assessment of the level of mental involvement is traditionally performed using dedicated evaluation grids and self-assessment questionnaires. This approach, besides raising problems related to wording and context, reflects a subjective evaluation based on the perception of subjects and their ability to express it faithfully and carefully [1,5]. Thus, recently, alternative assessment methods, relying on biosignal analysis, are being rapidly asserted. Usable biosignals are galvanic skin response (GSR), heart-rate variability (HRV), or electroencephalogram (EEG). Containing by its nature a wider range of information about the mental state than other biosignals, EEG is considered the most suitable for detecting and assessing mental involvement. In addition, it has the advantages of economic suitability, low invasiveness, and high temporal resolution [1]. Thus, in this work, we focus on EEG-based mental involvement detection.

EEG measures the voltage fluctuations resulting from ionic currents within neurons firing synchronously. Thus, EEG is the direct recording of brain electrical activity happening noninvasively at the scalp level. Scalp EEG electrode placement has been standardized to guarantee inter-subject and intra-subject comparability in both clinical and research settings [6]. Electrode localizations are defined as percentages distant from cephalometric landmarks [6]. An acknowledged method for low-density EEG electrode placement is the 10–20 system (19 electrodes) [6,7]. The standard implies the positioning of electrodes along fundamental ideal lines (antero-posterior, medial and lateral sagittal lines, frontal, central and parietal coronal lines) drawn starting from fixed landmarks: the inion (external protuberance of the occipital bone), the nasion (small depression immediately above the nose) and the preauricular points. The distance between one electrode and another is 10% or 20% of the total length of the line, hence the name of the system. The position of each electrode is labelled according to the lobe, or more in general the area of the brain, from which it is acquiring data: pre-frontal (Fp), frontal (F), temporal (T), parietal (P), occipital (O), and central (C). Moreover, electrodes are also numbered according to the side of placement: even-numbered electrodes are placed on the right hemisphere, while odd-numbered ones are placed on the left hemisphere. The presence of a “Z” in the electrode label means that it is placed in the middle sagittal plane [8]. The 10–20 system has undergone several extensions (e.g., the 10–10 system) up to the definition of a high-density electrode array. Indeed, spatial resolution is variable: from a low-density EEG montage, implying 19–25 electrodes, to a high-density EEG montage, implying a minimum of 64–256 channels [6,9]. On the other hand, wearable/portable systems with less than 19 electrodes have been studied due to their benefit of ease of use, especially in the brain computer interface (BCI) field of application, allowing researchers an inexpensive alternative to laboratory-based systems [5,10,11]. EEG is used to analyze subjects’ brain status and behavior based on frequency ranges of the signal, categorized into five human EEG frequency bands (or brainwaves): delta, theta, alpha, beta and gamma [12]. These widely recognized frequency bands are distributed along the EEG frequency band (0.1–100 Hz), even if the frequency range thresholds pertaining each band are not uniquely standardized. Roughly, delta, theta, alpha, beta and gamma bands are located lower than 4 Hz, between 4 Hz and 8 Hz, between 8 Hz and 12 Hz, between 12 Hz and 35 Hz, and higher than 35 Hz, respectively. They are associated with brain status, and thus are exploited to characterize it. Usually, the delta band is associated with sleep and dreaming, the theta band is associated with deep relaxing and drowsiness, the alpha band is associated with resting and passive attention, the beta band is associated with active mind and external attention, and gamma is associated with concentration [13]. Some studies also identify an intermediate band between alpha and beta, indicating sensory-motor rhythm (12–15 Hz), collocating beta bands between 15 Hz and 35 Hz [14]. Furthermore, some authors consider sub-sections within the frequency contents pertaining the EEG frequency bands, usually labelling them with progressive numbers corresponding to the ordered subsections (e.g., beta_1 is the first part of the beta frequency content). As with the definition of EEG bands’ frequency limits, the subsection frequency limits are not uniquely set.

Adopting the classification approach recently (2019) proposed by Wang et al., involvement indexes based on the spectral power (energy) of EEG-derived brainwaves can be categorized into basic and ratio ones [15]. The basic indexes can be defined as the power or relative power of single brainwaves, while the ratio indexes can be defined as the ratio of powers and/or of power summations, thus including more than one single brainwave, for more comprehensive indicators that more reliably reflect mental involvement. Indeed, although the literature agrees in attributing to the spectral power of each brainwave a close association with the cognitive patterns and mental state of subjects, this cannot always faithfully reflect variations of mental involvement [15]. In this context, an approach based on the ratio of spectral power of EEG-derived brainwaves seems to be preferable. The nomenclature indicating these kinds of EEG indexes is similar, but not always unique. In 1995, Pope et al. talked about “engagement index” [16], referring to a relationship among brainwaves reflecting the level of mental involvement in a task. More recently, in 2015, Coelli et al. referred to the same kind of index as “brainwave-based engagement index” [2]. In order to propose a unique nomenclature, the present systematic review analyzes the scientific material published in the literature regarding mental involvement indexes (explicitly defined in this way or not) computed as ratio indexes. Specifically, ratio indexes are meant to be ratios of functions involving the power spectral densities of more than one brainwave assessed via noninvasive EEG. Studies of interest have to consider EEG acquired on healthy populations in a psychological state not altered by external factors. The final aim is to address four main objectives, finding answers to the following research questions:

- What are the spectral EEG ratio indexes for mental assessment and how are they defined?
- How are they used in relation to the specific field of application, i.e., in relation to the subject’s activity?
- Is the spatial density of the EEG system used crucial for their computation?
- Are some electrodes more appropriate for their computation?

## 2. Materials and Methods

This systematic review was carried out and its findings presented according to the Preferred Reporting Items for Systematic Review and Meta-Analyses (PRISMA) guidelines, updated in 2020 [15].

### 2.1. Literature Search Strategy and Design

The literature search of peer-reviewed scientific research studies (referred to as documents in the following) was conducted in the period from January to March 2023 in four electronic bibliographic repositories: PubMed, Scopus, Web of Science, and IEEE Xplore. The literature was organized dividing the topic of interest (i.e., mental involvement indexes based on EEG-derived brainwaves) into the four areas of which it is essentially composed. Indeed, in order to reach our goal, we had to focus on: (1) the different possible tasks that the brain controls; (2) the type of EEG; (3) EEG characterization through brainwaves; (4) objective assessment of brain involvement using indexes derived from brainwave-related features. Based on these four search areas, the inclusion search expressions/terms/term roots (which are referred to as ‘keywords’ in the following) were:

(1) movement* control, movement* response, movement* task*, motor control, motor task*, motor response, sensory control, sensory task*, sensory response, emotion*, mental state*, mental effort, mental fatigue, mental task*, mental load, mental function*, cognitive load, cognitive task*, cognitive effort, cognitive fatigue, cognitive function*, stress, attention, vigilance, working memory, language task*, language processing, language control;
(2) EEG, electroencephalogra*;
(3) wave*, frequency band*, oscillation*, rhythm*, frequency content, frequency range;
(4) index*, indice*, characterization, marker*;

Specifically: ‘movement* control’, ‘movement* response’, ‘movement* task*’, ‘motor control’, ‘motor task*’, and ‘motor response’ were used to explore and include documents related to movement; ‘sensory control’, ‘sensory task*’, ‘sensory response’, and ‘emotion*’ were used to explore and include documents related to sensory and emotional aspects; ‘mental state*’, ‘mental effort’, ‘mental fatigue’, ‘mental task*’, ‘mental load’, ‘mental function*’, ‘cognitive load’, ‘cognitive task*’, ‘cognitive effort’, ‘cognitive fatigue’, ‘cognitive function*’, ‘stress’, ‘attention’, ‘vigilance’, ‘working memory’, ‘language task*’, ‘language processing’, and ‘language control’ were used to explore and include documents related to mental strain, including verbal/reading processing and temporal retention of information; ‘EEG’ and ‘electroencephalogra*’ were used to explore and include documents taking into consideration the EEG as signal of interest; ‘wave*’, ‘frequency band*’, ‘oscillation*’, ‘rhythm*’, ‘frequency content’, and ‘frequency range’ were used to explore and include documents characterizing EEG through brainwaves; ‘index*’, ‘indice*’, ‘characterization’, and ‘marker*’ were used to explore and include documents defining/using indexes. 

Inclusion keywords within each search area were combined through the Boolean operator ‘OR’, and in turn the resulting four inclusion keyword combinations were arranged through the operator ‘AND’ to create the search query. Moreover, in order to explore only documents pertaining to healthy human beings engaged in a continuative task and whose attention was not tested through stimuli, some exclusion keywords were also considered. Particularly, the following keywords were excluded: ‘animal*’, ‘stimul*’, ‘disease*’, ‘disorder*’, and ‘impairment’. The exclusion keywords were arranged in the search query through the operator ‘AND NOT’. The search field was limited by the application of the search query to the two fields of title and abstract concurrently. Eventually, two automatic filters about language and document type were performed on the search query: non-English documents, as well as reviews (including conference reviews) and book chapters, were filtered out from the search. No additional filters were performed on the publication date. The same search query was used to systematically look for documents in the four electronic bibliographic repositories. The specific forms used for each of them are reported in Appendix A.

### 2.2. Selection of Documents

All documents resulting from the application of the search query in Scopus, PubMed, Web of Science and IEEE Xplore were imported into the citation management tool Mendeley. There, duplicated documents were identified and discarded. The documents were further manually screened to exclude reviews, book chapters and non-English documents that may have remained after the automatic screening. Documents for which the abstract was not available were neglected. The screening of documents was performed with the purpose of including studies that considered:

- healthy human subjects, where the condition of health is intended especially in relation to the absence of neurological and/or psychiatric disorders or alteration conditions;
- awake subjects;
- subjects not under the effect of drugs or mind-altering substances in general (i.e., alcohol, tobacco, caffeine, etc.);
- non-invasive EEG used for assessment of mental state;
- EEG characterized in the frequency domain through the brainwaves;
- frequency bands of EEG brainwaves characterized only by the power spectral density of the signal, specifically: the power spectral density of the delta frequency band (from this moment indicated as δ); the power spectral density of the theta frequency band (from this moment indicated as θ); the power spectral density of the alpha frequency band (from this moment indicated as α); the power spectral density of the sensory-motor rhythm (from this moment indicated as SMR); the power spectral density of the beta frequency band (from this moment indicated as β); the power spectral density of the gamma frequency band (from this moment indicated as γ). Power spectral density could be also expressed as sum or mean over different EEG channels;
- the involvement spectral EEG ratio index resulting from the ratio between the functions of power spectral densities of some EEG brainwaves, involving more than one brainwave and being different at numerator and denominator.

In particular, based on this purpose, the following exclusion criteria were applied:

- studies involving animals;
- studies involving patients (subjects affected by a disease);
- studies involving subjects in a possibly altered state;
- studies involving sleeping subjects/patients;
- studies not considering noninvasive EEG;
- studies where EEG is not characterized according to brainwaves (delta, theta, alpha, sensory-motor rhythm, beta, gamma);
- studies where brainwaves are characterized by means other than power spectral density (δ, θ, α, SMR, β, γ);
- studies not considering spectral EEG ratio indexes based on the spectral power of EEG-derived brainwaves, expressed as in the mathematical form of Equation (1):

(1) $$SRI=\frac{f_{1}\left(a\cdot \delta ,\;b\cdot \theta ,\;c\cdot \alpha ,\;d\cdot SMR,e\cdot \beta ,\;g\cdot \gamma \right)}{f_{2}\left(h\cdot \delta ,\;i\cdot \theta ,\;j\cdot \alpha ,k\cdot SMR,\;l\cdot \beta ,\;m\cdot \gamma \right)}$$

where SRI stands for spectral ratio index, $f_{1}$ and $f_{2}$ are at least first-order in one of the power spectral densities, a, b, c, d, e, g, h, i, j, k, l, m are binary numbers (equal to 1 or 0) in any possible combination (except all equal to 1 that gives SRI=1). 

The document exclusion process was performed using the “or” operator logic among the established criteria. After title and abstract were screened sequentially according to the same criteria, documents that met the exclusion criteria were progressively discarded. In case neither the title nor the abstract contained necessary information for the screening evaluation, the same criteria were applied in the following phase of full-text review. Documents for which the full text was not available were neglected. Documents that did not meet the exclusion criteria were included in the review for the following evaluation steps.

Finally, the references of the documents included were screened in order to search for further relevant documents to be added, using the same procedure for exclusion used to select the included documents.

### 2.3. Collection of Information

All included documents were sorted based on publication year and their distribution over time was evaluated. In addition, they were qualitatively classified by text screening according to the application field considered by each described study. The possible categories were: (1) documents related to movement, (2) documents related to sensory and emotional aspects, (3) documents related to mental strain.

Selected documents were also imported and analyzed using the spreadsheet software Microsoft Excel. Each document was described in terms of studied population characteristics (size, gender, age, preferred hand), as well as in terms of EEG acquisition device characteristics (name, number of electrodes used excluding ground and reference, electrode montage). Referring to population characteristics, continuous features (i.e., age) were expressed according to the reporting modality of the reference document (e.g., as mean ± standard deviation, as median [interquartile range], as minimum–maximum range, or as mean and standard error), while categorical features (i.e., gender and preferred hand) were reported as absolute number, ratio, or percentage. If the information was not available, ‘NA’ (standing for ‘not available’) was indicated. The reported population size was the first indicated by the document, so that exclusion criteria eventually applied throughout the described study were not considered. Age and gender were reported accordingly, if possible (otherwise specified).

The electrodes used for the computation of EEG ratio indexes were specifically described in their number, positioning, and modality of use in the index formula (e.g., singularly or combined as sum or mean), if this information was available. The electrode use rate was then represented through a colored topographical bidimensional scalp map showing a different color or intensity of color based on frequency. 

From all included documents, a list of the EEG ratio indexes used was created, together with an indication of the specific documents that use them in the basic form or as derived indexes. Derived indexes are defined here as indexes using the basic form of an original index but calculated over subsections of wave frequency bands using the introduction of weights into the terms of the formula, or using a formula where the terms are the original index (as in the case of the indexes defined as reciprocals of the basic form). Eventually, indexes were evaluated for their frequency of use and field of application, based on the application field of documents where they were considered.

### 2.4. Quality Appraisal

The quality and risk of bias of the selected documents were appraised through the Joanna Briggs Institute tools [15], using the appropriate checklist according to the study design [17]. Several items pertaining the study described in each document were labelled with “yes”, “no”, “unclear”, or “not applicable” based on the modality of reporting the related information. The quality appraisal was assessed independently by four authors and possible discrepancies resolved by all authors after aggregated revision and discussion. Eventually, the overall score assigned to each document was assessed via the total amount of affirmative answers, expressed as a percentage with respect to the full score, given by the number of evaluated items. No exclusions were performed based on the quality appraisal.

Limitations of the selected documents were estimated based on the specifications of the electrodes used to characterize the EEG bands, then combined to compute the mental involvement index.

## 3. Results

From the application of the search query, 1366 documents resulted: specifically, 526 in Scopus, 407 in PubMed, 378 in Web of Science, and 55 in IEEE Xplore. Then, 706 documents were automatically identified as duplicated and discarded, so that 660 remained for further screening. After manual screening 626 documents remained. Based on the title evaluation, 109 documents were excluded. All the resulting documents had abstracts available. Based on the abstract evaluation, 47 documents were excluded. Where necessary and if available (24 documents had text not available), the full text was evaluated. Among the evaluated documents, 4 were studies involving animals, 38 were studies involving patients, 6 were studies involving subjects in possibly altered states, 43 were studies not considering only noninvasive EEG, and 318 were studies not considering spectral EEG ratio indexes (e.g., considering only single brainwaves). After full text analysis, 37 documents were recognized as not compliant with the established exclusion criteria and were selected for inclusion in the review [2,18,19,20,21,22,23,24,25,26,27,28,29,30,31,32,33,34,35,36,37,38,39,40,41,42,43,44,45,46,47,48,49,50,51,52,53].

The cited references of the included documents totaled 1609. Of them, 115 documents were automatically identified as duplicated and discarded, so that 1494 remained for further screening. After manual screening, 1319 documents remained. Based on the title evaluation, 676 documents were excluded. A few documents (4) had abstracts not available. Based on the abstract evaluation, 444 documents were excluded. Where necessary and if available (47 documents had text not available), the full text was evaluated. Among the evaluated documents, 1 was a study involving patients, 2 were studies involving sleeping people, 16 were studies not considering only noninvasive EEG, and 84 were studies not considering spectral EEG ratio indexes (e.g., considering only single brainwaves). After full text analysis, 45 documents were recognized as not compliant with the established exclusion criteria and were selected for inclusion in the review [5,16,54,55,56,57,58,59,60,61,62,63,64,65,66,67,68,69,70,71,72,73,74,75,76,77,78,79,80,81,82,83,84,85,86,87,88,89,90,91,92,93,94,95,96]. 

**Figure 1:**
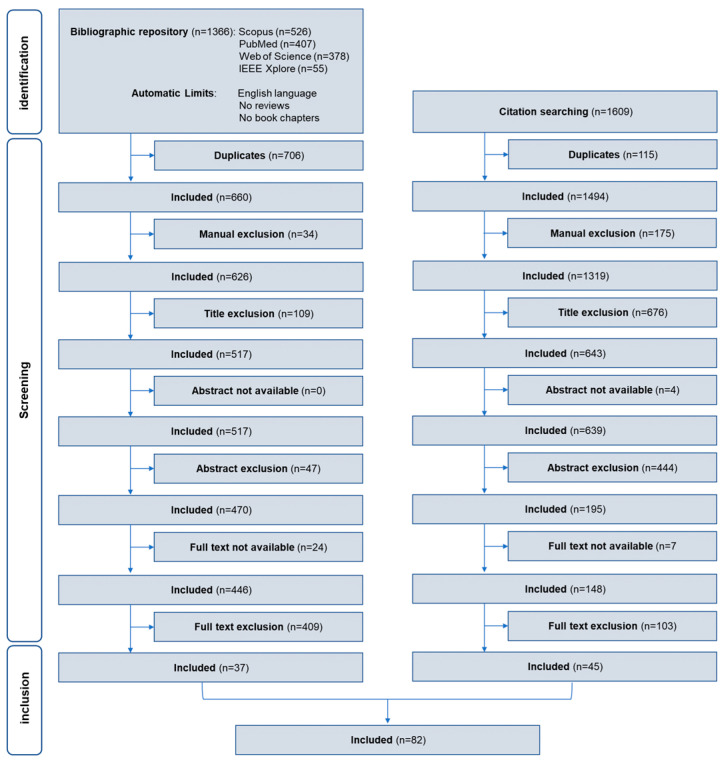
Flow diagram of the systematic review showing the design of the literature and citation searches for identification of eligible documents, of the screening criteria and selection.

Overall, from the application of the search query and the citation search, 82 documents were included in the review. Figure 1 shows in detail the literature and citation searches, as well as the screening phases, that led to the selection of the documents included in the review.

**Figure 2:**
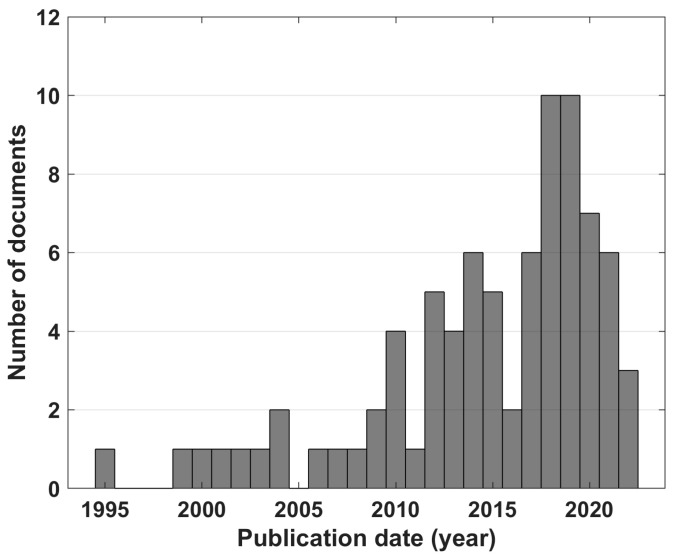
Distribution of the publication years of documents considering involvement indexes.

Most of the included documents were articles (specifically 65, 79%) [5,16,18,19,20,21,22,23,26,27,28,29,30,31,32,33,34,35,36,37,38,39,40,42,43,44,46,47,52,53,54,55,56,57,58,59,60,62,63,64,65,66,67,68,69,70,71,72,73,74,75,76,77,78,79,80,81,84,86,88,89,90,91,92,96], while the remainder (specifically 17, 21%) were conference papers [2,24,25,41,45,48,49,50,51,61,82,83,85,87,93,94,95]. Overall, the included documents covered the time period from 1995 to 2022. About 44% of included documents were published in the last five years, with 24% of documents published between 2018 and 2019. Figure 2 shows the distribution of document publication dates expressed in years.

**Figure 3:**
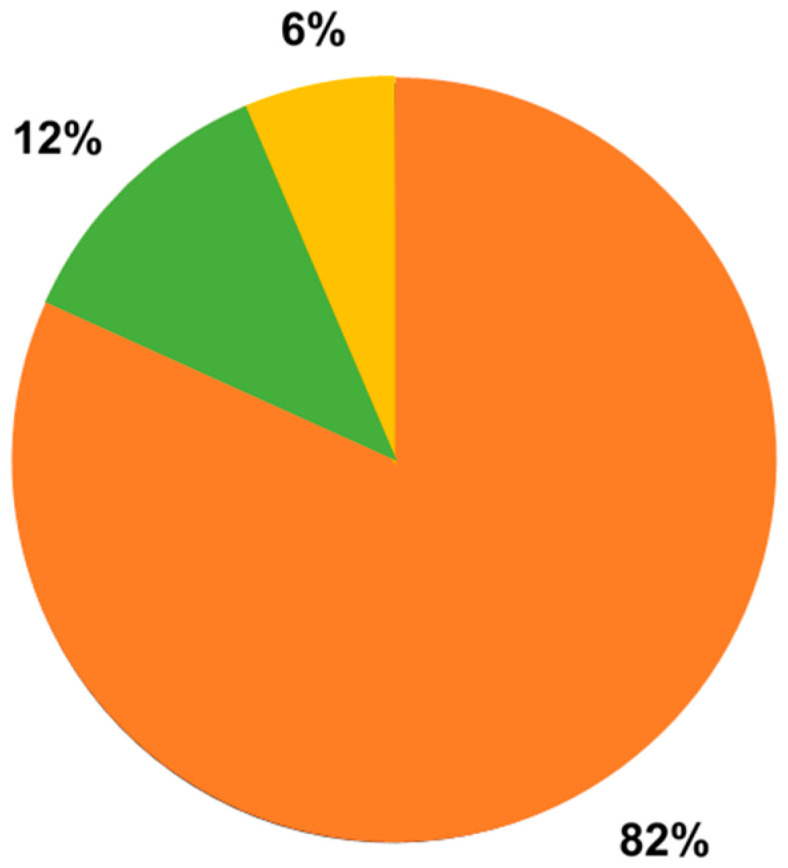
Distribution of documents included based on the application field: 5 documents related to movement ([20,21,23,58,60]), 10 documents related to sensory and emotional aspects ([19,26,40,42,43,51,54,61,84,94]), 67 documents related to mental strain (the remaining included documents).

In Figure 3 a classification of the documents based on the application field is proposed. The majority (82%) were classified as related to mental strain, half of them pertaining to learning (e.g., scholars), driving and working contexts (about 19%, 16% and 13%, respectively). The remaining were classified as related to sensory and emotion aspects (12%) or to movement (6%).

Table 1 includes the characteristics of the studied populations. The population size was not specified for three documents, while among the other 79, 50% enrolled a population including less than 20 individuals, 28% enrolled a population including 20 to 40 individuals, 14% enrolled a population including 40 to 60 individuals, and only 8% enrolled a population including more than 60 individuals. The population gender was not specified for 23 documents, while among the other 59, 65% of enrolled individuals were male. The population age was not specified for 14 documents, while among the other 68, one document enrolled only children (5–10 years old) [82], one document enrolled only adolescents (10–19 years old) [34], 55 documents enrolled adult individuals (19–60 years old), one document considered only over-60 individuals [46], and the remaining enrolled a mixed-age population. The population’s preferred hand was only specified in 16 (20%) and in these documents the whole population was right-handed. Results for quality appraisal of all selected documents are also reported in Table 1.

Table 2 includes the characteristics of the EEG acquisition devices. The device most used was the Emotiv Epoc System, employed in 19 (23%) included documents. The number of electrodes acquired was not specified in two documents, while the montage was low-density in 13% of documents, high-density in 6% and very-low-density in the remaining documents. The electrode montage system (EMS) was not specified in eight documents, while in the other 74 the international 10–20 system (or its extended versions) was employed. In Table 2, not only the number of acquired channels (NAC) is indicated, but also the channels used in the computation of EEG ratio index formulas (channels used for power analysis, CPA). If the modality through which the acquired channels were combined in the computation of the index (e.g., singularly and differently for different EEG bands, or as sum or average) was specified, the ‘channels specified in formula’ (CSF) item was labeled with a checkmark. 

**Figure 4:**
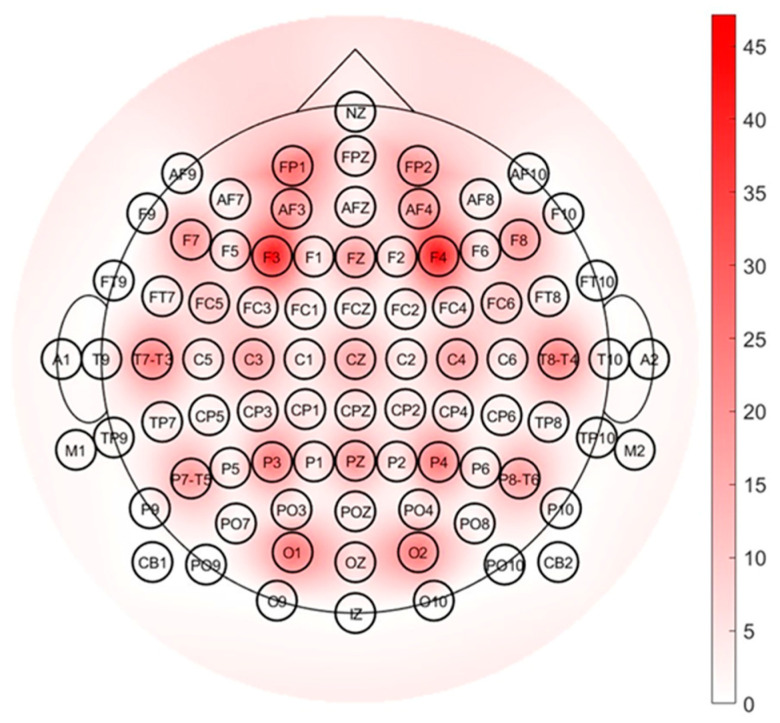
Topographical bidimensional scalp map showing the usage rate of electrodes; a standard montage system including 81 electrodes is considered.

Figure 4 shows a topographical bidimensional scalp map specifically considering an extended 10–20 EEG electrode montage system, where each electrode is characterized in terms of the number of times it is considered in the literature to compute the involvement index. In particular, the range of colors from white to red corresponds to a higher use rate of electrodes. From the figure it is possible to conclude that the most used electrode positions for computation of involvement indexes are F3 and F4, followed by Fz and FP1, i.e., the frontal and prefrontal areas. 

In Table 3 a list of the EEG ratio indexes used is reported. Overall, we found 37 different formulations of involvement indexes. Besides the basic form, the specific documents that use each index, in the basic form or as derived indexes, is indicated. Documents are reported already classified based on their application field.

The analysis performed on included documents regarding indexes showed that several times the indexes were normalized through a logarithm [62,66,69,73,84,90]. Moreover, derived indexes were used for I_1_, I_2_, I_3_, I_4_, I_5_, I_8_, I_9_, I_11_, I_16_, I_18_, I_21_, I_24_, I_25_, I_29_, and I_34_.

Index I_1_, besides its basic form, is also used in derived forms: as I_1_(left)–I_1_(right) (where ‘left’ and ‘right’ refers to the position of electrodes on the two hemispheres); as α/β_high (where β_high is the subsection of β in the frequency range 25–30 Hz, [51]); and as reciprocal (1/I_1_). Furthermore, from I_1_ and its reciprocal form two involvement indexes, often defined as ‘arousal index’ and ‘valence index’, are derived, specifically considering electrode positions F3, F4, AF3, AF4. The possible forms of ‘arousal index’ and ‘valence index’ are: $I_{1}\left(F3+F4\right)$ (arousal); $I_{1}\left(AF3+AF4+F3+F4\right)$ (arousal); $1/(I_{1}\left(AF3+AF4+F3+F4\right)$) (arousal); $1/I_{1}\left(F4\right)-I_{1}\left(F3\right)$ (valence).

Index I_2_, besides its basic form, is also used in derived forms: β_II_/(α + θ) (where β_II_ is the subsection of β in the frequency range 20–50 Hz, [45]); using weights in the terms ((0.4∙α + 0.6∙θ)/0.5∙β and (0.6∙α + 0.4∙θ)/0.5∙β, [50]); and as reciprocal (1/I_2_).

Index I_3_, besides its basic form, is also used in derived forms: β_1_/θ (where β_1_ is the subsection of β in the frequency range 16–20 Hz, [34]); as reciprocal (1/I_3_).

Index I_11_, besides its basic form, is also used in the derived form (θ + α)/10∙γ_1_ (where γ_1_ is the subsection of γ in the frequency range 31–39.75 Hz, [39]).

Indexes I_4_, I_5_, I_9_, I_16_, I_18_, I_24_, I_25_, I_29_, and I_34_, besides their basic forms, are also used as reciprocals (1/I_4_, 1/I_5_, 1/I_9_, 1/I_16_, 1/I_18_, 1/I_24_, 1/I_25_, 1/I_29_, 1/I_34_).

Indexes I_8_ and I_21_ were found only in their derived forms: (α + β)/δ_1_ (where δ_1_ is the subsection of δ in the frequency range 0.5–2 Hz, [65]) and $\left(SMR+\beta_{middle}\right)/\theta$ (where $\beta_{middle}$ is the subsection of $\beta_{middle}$ in the frequency range 16–20 Hz in [70], while it is not specifically defined in [95]).

Table 2, together with Table 3, make evident that the same indexes are not always computed considering the same electrodes. Moreover, sometimes the electrodes considered and the modality through which they were combined (e.g., singularly and differently for different EEG bands, or as sum or average) are not specified (or at least deducible) from the study description. The electrodes used for index computation were not specified in 25 documents (30%), while how they are managed within the formula (e.g., whether the EEG band spectral power is computed from one specific electrode, or from more electrodes that are then summed or averaged) is not specified in 46 documents (56%). 

**Figure 5:**
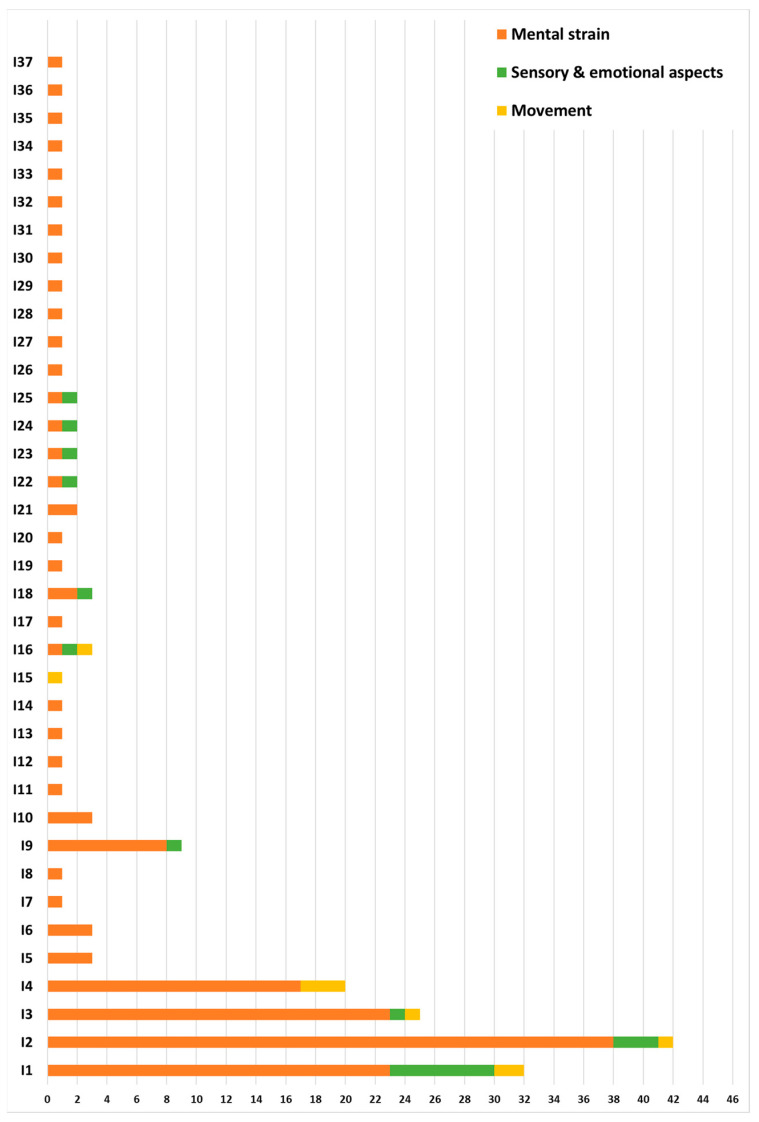
Use rate of involvement indexes.

Figure 5 shows the rate of use of each involvement index. In the computing of each index rate, the derived forms were included.

## 4. Discussion

The present review systematically examined the scientific literature about EEG-derived ratio indexes used to assess the mental involvement of healthy human subjects, in order to understand what they are, how they are defined and used, and what their best fields of application are. Overall, 82 documents were included, confirming intense scientific activity around this topic, considering that, based on the chosen exclusion criteria in the document selection, our review focuses on a subsector of EEG-based assessment of mental involvement. The first selection was based on the language and the document type, excluding non-English documents, reviews and book chapters. The first choice relies on the fact that the proper language of scientific literature is English. The exclusion of book chapters relies on the wish to focus on research published in scientific journals. The exclusion of reviews was consequent to a preliminary search in the literature and supported by it. The research involved the application of the same query presented in the ‘Literature search strategy and design’ section, but including only the reviews: they were evaluated one by one to test the novelty of our approach [97,98,99,100,101,102,103,104,105,106,107]. Since they were not in line with the objectives of our research, we confirmed the utility of the present systematic review and we decided to exclude them from the research. No limits were imposed for the publication year, since an evaluation of the time trend for the use of involvement indexes was among our purposes. As indicated by Pope et al., which was the first document on our chronologically sorted list of documents (published in 1995), the first literature on EEG-based assessment of attention and vigilance dated back to around 1990. As a consequence, before the evaluation of the definition and use of EEG-based ratio indexes, we felt a preliminary observation on their applicability nowadays was important, and Figure 2 clearly shows that, throughout the years, there is a growing use rather than a decommissioning of these kinds of indexes. 

The exclusion criteria were defined based on the objectives of the review. The choice to include only human studies relies on the wish to collect documents describing scientific studies carried out in real scenarios (or controlled conditions faithfully reproduced in laboratory contexts), therefore not based solely on physiological knowledge, as studies carried out using animal experiments could be. The decision to include only healthy subjects with no known pathologies is linked to the fact that pathologies, especially ones affecting the nervous system, could interfere with the physiological behavior of the brain with respect to mental involvement. These conditions, as well as alterations in the psychological state of the subject resulting from the intake of external substances, could represent a bias in the correct interpretation of the functioning and role of the mental involvement index considered. Therefore, these should be particular conditions of interest to be evaluated in dedicated reviews. Furthermore, the conscious involvement of an individual occurs while the subject is awake and not during sleep; therefore, studies on awake populations were considered more suitable. The decision to focus on power spectral density depended on the fact that it is one of the most traditional and consolidated brainwave characterization features in terms of use and meaning. Finally, the choice to focus on studies that considered the class of ratio EEG indexes is justified by the fact that different brainwaves can better emphasize the brain condition of the subject than a single one. 

Going deeper on this last aspect, the literature showed that it is possible to identify three classes of indexes on the EEG: basic, burst and ratio ones. The basic indexes are the relative power of the EEG frequency bands (i.e., the spectral power of each EEG frequency band, δ, θ, α, β, and γ, normalized by the sum of all EEG frequency bands). The burst indexes are set by counting the number of peaks over a certain threshold in the EEG frequency band time trend. The ratio indexes are essentially defined as a ratio of basic indexes [78]. In the approach of Pope [16], indexes coming from the same EEG frequency band were also considered (specifically, α(T5 + P3)/α(Cz + Pz) and α(O1)/α(O2)), and other authors use as involvement indexes the basic ones [27,46,68,78], but in the literature a combined-brainwave approach seems to be preferred and more used [19,78]. In addition, the same Pope study concluded that index I_2_ (including three EEG bands) reflected mental involvement in the considered context (working context) better than the other index considered (i.e., I_1_, including two EEG bands, α(T5 + P3)/α(Cz + Pz) and α(O1)/α(O2), both including only one EEG band) [16]. Thus, an integrated approach should highlight a clearer recognition of mental involvement, since, in general, more intense high-frequency EEG band power (α, β, and γ) and suppressed low-frequency EEG band power (δ and θ) are both related to growing cognitive abilities [60]. 

In order to verify if some indexes are more used in particular contexts than in others, documents were classified into three main categories, the same ones considered when searching for proper keywords composing the research query: one context was related to the influence of performing physical activity on mental attention and vigilance (synthetically labelled as “movement”), another was related to the assessment of emotions and sensory perception (synthetically labelled as “sensory and emotional aspects”), and the last was related to mental strain, implying a brain status of attention and vigilance, and including information processing and retention, for example, for the management of learning and working tasks (synthetically labelled as “mental strain”). Most of the documents included fell into the latter category, as there is an increasing interest from a research point of view in monitoring these aspects which, if excessive in daily life, can have negative consequences on the quality of life of human beings. We would like to point out that, strictly speaking, a document could cover several categories, since all the documents included fall within the common theme of mental involvement, which often implies a mixture of cerebral reactions such as mental fatigue and the onset of emotions. Our definition of categories was based on the predominant aspect of each study, without the pretension of rigidly classifying each study, because classes inevitably overlap. Therefore, we speak of qualitative classification.

Approximately half of the included documents enrolled in their studies small populations (less than 20 individuals), and this could prevent the possibility of generalization of the obtained results. Statistical analyses were often made possible considering different epochs throughout the experiment duration. Nevertheless, as Chen et al. observed, each person has a subjective brain reaction while performing a task involving movement/emotion/high vigilance [87]. Thus, finding a single index that can be used to monitor mental involvement of a whole population is quite impossible. This partially justifies the use of many different formulations (37) of the involvement indexes. The included documents also revealed a small number of studies on specific age ranges. It would be interesting to have population stratification of brain behavior based on the phases of life, which could be very different, especially at the extremes, i.e., during the developmental age (up to about 20 years) and in the elderly (over 60). Another relevant aspect that seems to be neglected in the included documents is the consideration of brain lateralization, of which handedness is the most evident reflection [20]. The left and right hemispheres of the human brain are dedicated to different types of information processing, and much of our cognitive abilities are lateralized more to one side or the other. Only 20% of included documents specified the preferred hand, and among them, the whole population was right-handed. Thus, we decided not to introduce an exclusion criterion based on handedness since the counterpart (left-handed population) was in any case not represented.

Most of the used EEG acquisition devices were very-low-density or partially exploited the available electrodes of the acquisition system. This is a remarkable aspect because the application of involvement indexes is in general in daily life context, and thus testing acquisition systems that can be easily accustomed outside the clinical and laboratory environment is very useful. An example is the Emotiv Epoc System, used in approximately 40% of the involvement index-related documents included here from the last five years, although the market continues to offer many wearable and user-friendly solutions in a healthcare system that is increasingly evolving towards telemedicine. 

The electrode positions used most frequently for computation of involvement indexes are in the frontal and prefrontal lobes. They are selected especially when very few electrodes are used for acquisition or when only some of the acquired electrodes are used for involvement index computation. This is in accordance with what is known about frontal/prefrontal neurology in the literature. Indeed, prefrontal areas are associated with attentional and cognitive skills [66] and frontal areas seem to be related to analytical thinking, decision making and problem solving [25,38]. Particularly, the role of the prefrontal cortex is guaranteed by its connection with other regions and its ability to integrate different information sources, memory, and sensory systems [108]. Therefore, in an attempt to limit the number of electrodes used, employing only the minimum necessary ones, literature knowledge on neurophysiology confirms and validates the decision to calculate the indexes of involvement in these areas of the cortex.

Among the many (37) indexes that were extracted from examining the 82 included documents, the most frequently used are I_1_ (β/α), I_2_ (β/(α + θ)), I_3_ (β/θ), and I_4_ (θ/α), including their derived forms. Many times, the involvement indexes are used through the reciprocal formula. We decided to ascribe them the same role as the original formulas since the information content is essentially the same. On the other hand, derived indexes given by the subtraction of the same index computed at the left and right brain hemispheres are related to evaluation of the asymmetry of the index, which may be justified by lateralization of the brain. 

From evaluation of the included documents, we cannot outline a development trajectory or clear direction towards the use of certain indexes rather than others because, even if some documents define new ratio indexes (e.g., [18]), the more traditional ones (I_1_, I_2_, I_3_) continue to prove efficient. The most frequent indexes (I_1_, I_2_, I_3_), even if introduced about 30 years ago, show increasing use over the years, faithfully reflecting the growing publications around this topic, while the less used (and new) indexes were mostly introduced around 2018/2019. 

This review pointed out research gaps in the lack of standardization of the definition of the frequency limits of EEG brainwaves (as well as their subsections), and in the identification of the scalp area where EEG brainwaves have to be extracted. Indeed, while there is standardization in the electrode montage through the international 10–20 system, which is used by almost all the included documents, an analogous standardization was not found in the definition of the extremes of the frequency band, and each researcher used his/her own approach. Moreover, many times, it is not specified which electrodes are considered for computation of the index, or if specified, the authors did not specify how the power spectral densities of the extracted EEG brainwaves are combined (e.g., as sum, as mean, etc.). These aspects prevent a real comparison among studies using the same involvement index and, possibly, show different performances. Only when definition of the indexes is unique in the description of the involved EEG brainwaves and in the considered electrodes and following phases of EEG brainwave extraction and index computation, a comparison among studies will be possible, allowing identification of the most suitable and accurate indexes, possibly also in relation to the field of application. Besides these observations, from this systematic review of the literature we cannot deduce that one index is more used or more proper for a particular field of application, since indexes I_1_–I_4_ were used in all those considered here. The prevalence of their use in the mental strain class can be interpreted as due to a greater prevalence of studies in this category. 

Providing a quantitative measurement of the accuracy of the identified indexes is outside the aim of this review. Indeed, literature usually reports only the discriminatory power of the algorithms of classification to which these indexes are given as inputs and not the accuracy of the indexes per se.

Additionally, the use of some indexes involving the spectral power of high frequencies in the EEG band is also conditioned by the sampling rates of the acquisition devices. Indeed, as observed by Jebelli et al., wearable devices (to be preferred in involvement monitoring) have low temporal resolution [68] and this limits the EEG frequency content.

Eventually, after analyzing the use of the 37 ratio indexes based on spectral EEG brainwaves for the assessment of mental involvement reported here, it was not possible to identify the most suitable ones for specific fields of application, nor a unique calculation method for each of them, due to the lack of standardization. These findings highlight the gaps in the literature, which will hopefully be filled by further studies, given the relevance of the subject and the practical implications associated with it. 

The main limitation of the present review is its reliance on the selection of only ratio indexes based on spectral EEG brainwaves for the assessment of mental involvement. Indeed, in the literature there are several kinds of involvement indexes and the ones considered here are the most standard, and are supposed to amplify the differences among the physiological statuses associated with single EEG brainwaves [78], which allowed us to make in-depth evaluations, but do not necessarily represent the optimal ones. 

## 5. Conclusions

The present systematic review provided an insight into the spectral EEG ratio indexes used by scientific material published in the literature to assess mental involvement. A standardization in the definition of these indexes is missing, both in the considered frequency bands and in the exploited electrodes. Future research may focus on the development of indexes with a clear and unambiguous definition in order to guarantee the reproducibility and comparison of studies that use them to assess, monitor and characterize mental involvement. Possibly, it will then be possible to define the best indexes for a specific application and context.

## Figures and Tables

**Table 1:** Studied population characteristics.

Ref.	QA ^1^	Pop. Size ^2^		Gender (M/F) ^3^		Age (Years) ^4^	Preferred Hand ^5^
[2]	75%	9		8/1		24 ± 2.9 [18–28]	R: 9 L: 0
[5]	88%	30		10/20		AVG: 20.87 [18–43]	R:30 L:0
[16]	50%	6		NA		AVG: 35.5 [25–50]	NA
[18]	50%	10		10/0		NA	R: 10 L: 0
[19]	75%	30		16/14		[8–70]	NA
[20]	80%	14		6/8		M: 43.8 ± 9.2 F: 34.7 ± 11.9	R: 14 L: 0
[21]	50%	14		5/9		22.4 ± 1.6	NA
[22]	38%	4		4/0		[23–33]	R: 4 L: 0
[23]	50%	12		11/1		[21–35]	NA
[24]	25%	4		NA		[24–26]	NA
[25]	50%	4		NA		NA	NA
[26]	100%	42		31/11		20.81 ± 1.13	NA
[27]	80%	10		10/0		22.4 ± 1.7	NA
[28]	100%	16		16/0		23.1 ± 1.8	R: 16 L: 0
[29]	30%	20		20/0		AVG: 27	R: 20 L: 0
[30]	80%	17		11/6		AVG: 32.22; SE: 2.2 [21–40]	NA
[31]	88%	10		7/3		20.6 ± 3.2	R: 10 L: 0
[32]	40%	35		35/0		20.9 ± 1.57	R: 35 L: 0
[33]	75%	57	(28 YA)	18/39	12/16	25.39 ± 3.03 [21–31]	R: 57 L:0
			(29 O)		6/23	70.17 ± 3.38 [65–78]	
[34]	50%	29		17/12		[10–13]	NA
[35]	63%	14		8/6		20 ±1.5 [17–21]	NA
[36]	69%	30		18/12		22.1 ± 1.77 [20–28]	NA
[37]	75%	11		NA		24 ± 2.2	NA
[38]	38%	30		19/11		AVG: 24 [18–43]	NA
[39]	38%	2		2/0		[20–29]	NA
[40]	50%	34		17/17		[19–30]	NA
[41]	38%	82		35/47		AVG: 26	NA
[42]	88%	8		8/0		36.6 ± 9.2	R: 8 L:0
[43]	38%	14		NA		NA	NA
[44]	38%	15		NA		[22–33]	NA
[45]	38%	5		NA		NA	NA
[46]	63%	105		NA		[60–80]	NA
[47]	63%	21		21/0		AVG: 40.1 [29–47]	NA
[48]	50%	6		NA		NA	NA
[49]	38%	41		NA		NA	NA
[50]	38%	2		NA		NA	NA
[51]	50%	19		17/2		NA	NA
[52]	50%	40		40/0		39.06 ± 7.75	NA
[53]	75%	45		45/0		AVG: 20	NA
[54]	50%	32		16/16		AVG: 26.9 [19–37]	NA
[55]	88%	39	32 (dataset of 54)	NA	16/16	AVG: 26.9 [19–37]	NA
			7		NA	37.9 ± 8.8	NA
[56]	100%	22		22/0		22.54 ± 1.53	NA
[57]	38%	44		NA		≥18	NA
[58]	88%	7		7/0		26.3 ± 1.9	NA
[59]	50%	64		64/0		[20–25]	NA
[60]	77%	30	15	27/3	13/2	22.42 ± 2.30	R:30 L:0
			15		14/1	23.67 ± 2.09	
[61]	63%	6		3/3		AVG: 30.16	NA
[62]	63%	74		36/38		NA	NA
[63]	75%	10		6/4		AVG: 27.5 [24–36]	NA
[64]	88%	20		16/4		28 ± 2	NA
[65]	63%	21		21/0		[25–35]	NA
[66]	54%	77		30/ 47		19.6 ± 2.5	NA
[67]	88%	10		NA		AVG: 22 [20–24]	NA
[68]	88%	11		11/0		37.9 ± 8.8 [26–50]	NA
[69]	69%	18		18/0		30.1 ± 10.8	NA
[70]	75%	7		7/0		[22–28]	NA
[71]	75%	40		26/14		[19–38]	NA
[72]	75%	10		10/0		[20–28]	NA
[73]	75%	36	20	NA	20/0	[19–22]	NA
			16		NA	NA	NA
[74]	100%	18		16/0		23.1 ± 1.4 [21–26]	R:18 L:0
[75]	38%	36		NA		[8–40]	R:36 L:0
[76]	88%	42		16/26		24.26 ± 1.17 [20–26]	R:42 L:0
[77]	100%	9		NA		[25–40]	NA
[78]	88%	NA		NA		AVG: 26	NA
[79]	54%	60		NA		[18–40]	R:60 L:0
[80]	75%	20		20/0		25.6 ± 2.56 [22–32]	NA
[81]	100%	50		NA		28.48 ± 2.63 [25–33]	NA
[82]	75%	24		10/14		7.56 ± 0.86 [6–8.5]	NA
[83]	38%	NA		NA		NA	NA
[84]	88%	10		10/0		[26–55]	NA
[85]	63%	30		15/15		22.3 ± 6.88 [18–57]	NA
[86]	88%	24		12/12		AVG: 25 [22–27]	NA
[87]	63%	5		NA		NA	NA
[88]	63%	15		8/7		21.8 ± 2.73 [20–27]	NA
[89]	100%	52		36/16		28 ± 10 [20–70]	NA
[90]	88%	110		27/83		29.34 ± 10.17 [18–55]	NA
[91]	88%	20		20/0		23 ± 4.40	NA
[92]	69%	18		12/6		23.1 ± 1.9	NA
[93]	50%	13		13/0		[21–30]	NA
[94]	50%	NA		NA		NA	NA
[95]	50%	10		5/5		NA	NA
[96]	46%	42		26/16		20.81 ± 1.13	NA

^1^ QA: ‘quality appraisal’; ^2^ Pop. Size: ‘population size’; YA ‘young adults’; O ‘older subjects’; NA: ‘not available’; ^3^ M/F: ‘male/female’; NA: ‘not available’; ^4^ Age values are reported in terms of mean ± standard deviation or as [range], based on the original studies. AVG: ‘average’; SE: ‘standard error’. When the gender is not specified the age values are the same for both genders; NA: ‘not available’. ^5^ R: right hand; L: left hand; NA: ‘not available’.

**Table 2:** EEG acquisition device characteristics.

Ref.	Device	NAC ^1^	CPA ^2^	EMS ^3^	CSF ^4^
[2]	NeuroScan	19	F4, F3, F7, F8, Pz, P3, Fz, C3, Pz, P3, P4, Cz, FP1	10–20	
[5]	Emotiv EPOC system	14	F3, F4, I2	10–20	
[16]	NA	7	Cz, Pz, P3, P4	10–20	✓
[18]	Emotiv EPOC system	14	NA	10–20	
[19]	BIOPAC (EEG100C)	1	NA	NA	✓
[20]	NEXUS-32	21	F3, Fz, F4, C3, Cz, C4, P3, Pz, P4, O1, O2	10–20	
[21]	NeuroScan	20	FP1, FP2, F7, F8, F3, F4, Fz, C3, C4, Cz, P3, P4, Pz, T3, T4, T5, T6, O1, O2, Oz	10–20	✓
[22]	EBNeuro	7	P4, F4	E 10–20	✓
[23]	MOVE system	64	NA	E 10–20	
[24]	NeuroSky’s Mind-Band	1	FP1	10–20	
[25]	NeuroScan	2	FP1, FP2	10–20	✓
[26]	Device of OpenBCI	15	FP1, FP2, F7, F3, Fz, F4, F8, T7, Cz, T8, P7, Pz, P8, O1, O2	10–20	✓
[27]	Device of mBrainTrain	24	FP1, FP2, AFz, F7, F3, Fz, F4, F8, T7, T8, C3, C4, Cz, CPz, M1, M2, P7, P3, Pz, P4, P8, POz, O1, O2	10–20	✓
[28]	NeuroScan	13	Fp2, Fp1, F4, F3, A2, A1, C4, C3, P4, P3, Fz, Cz, Pz	10–20	✓
[29]	Emotiv EPOC system	14	AF3, AF4	10–20	✓
[30]	Mitsar-EEG 201	20	Fz, Pz	10–20	✓
[31]	NA	22	NA	E 10–20	✓
[32]	MOBITA (wireless EEG)	27	Fp1, Fpz, Fp2, F7, F3, Fz, F4, F8, FC5, FC1, FC2, FC6, C3, Cz, C4, CP5, CP1, CP2, CP6, P7, P3, Pz, P4, P8, O1, Oz, O2	10–20	✓
[33]	BrainVision Recorder	32	NA	10–20	
[34]	NA	16	FP1, FP2, F3, F4, F7, F8, C3, C4, T3, T4, T5, T6, P3, P4, O1, O2	10–20	
[35]	Enobio	8	FP1, FP2, P3, P4, O1, C4, T7, T8	10–20	✓
[36]	NA	NA	NA	NA	
[37]	BIOS-S8	6	Fpz, Fz, Cz, Pz, C3, C4	10–20	✓
[38]	BrainVision	4	F3, F4, O1, O2	10–20	
[39]	MindSet	1	FP1	10–20	
[40]	Emotiv EPOC system	14	AF3, F7, F3, FC5, T7, AF4, F4, F8, FC6, T8	10–20	
[41]	NA	64	Fz, Pz, F3, F4, O1, O2	E 10–20	
[42]	Biosignalplux (EEG)	2	F3, F4	10–20	✓
[43]	Emotiv EPOC system	14	O1, O2, P7, P8, T7, T8, FC5, FC6, F3, F4, F7, F8, AF3, AF4	10–20	✓
[44]	Emotiv EPOC system	14	F3, F4, P7, P8	10–20	✓
[45]	Emotiv EPOC system	14	O1, O2, P7, P8, T7, T8, FC5, FC6, F3, F4, F7, F8, AF3, AF4	10–20	
[46]	NA	1	NA	NA	
[47]	eegoTMmylab	63	C1, C2, CP1, CP2, P1, P2	E 10–20	
[48]	mBrainTrain (SMARTING system)	NA	Fz, Cz, CPz, Pz	NA	
[49]	Wave Rider system	2	FP1, FP2	10–20	
[50]	BIOPAC (MP100)	2	NA	10–20	
[51]	Enobio device	8	Fp1, Fp2, F3, F4, T7, T8, Pz, P4	10–20	✓
[52]	NA	64	FP1	10–20	
[53]	Emotiv EPOC system	14	AF3, AF4	10–20	✓
[54]	Emotiv EPOC system	14	Fz, AF3, F3, AF4, F4	10–20	✓
[55]	Emotiv EPOC system	14	AF3, AF4, F3, F4	10–20	✓
[56]	Net Amps 300	128	FP1, FP2, F3, F4, F7, F8, C3, C4, T3, T4, T5, T6, P3, P4, O1, O2, Fz, Cz, Pz	10–20	✓
[57]	BIOPAC (EEG100A)	22	O3, O4, F3, F4	10–20	
[58]	NA	2	F3, F4	10–20	
[59]	Emotiv EPOC system	14	AF3, AF4, F3, F4	10–20	✓
[60]	BIOPAC (MP150)	20	NA	10–20	
[61]	Emotiv EPOC system	14	F3, F4	10–20	✓
[62]	BioSemi ActiveTwo	9	F3, Fz, F4	10–20	✓
[63]	BrainAmp	64	F3, F1, Fz, F2, F4, FC3, FC1, FCz, FC4, C3, C1, C2, C4, CP3, CP1, CPz, CP2, CP4, P3, P1, Pz, P2, P4, PO3, POz, PO4, O1, Oz, O2	E 10–20	
[64]	g.MOBIlab (EEG)	8	FC3, FC4, C3, C4, C5, C6, CP3, CP4	10–20	✓
[65]	NA	13	Fp1, Fp2, F3, F4, T3, T4, C3, C4, P3, P4, O1, O2, Cz	10–20	
[66]	BioSemi ActiveTwo	9	F3, Fz, F4	10–20	✓
[67]	VEEG1240	16	NA	10–20	
[68]	Emotiv EPOC system	14	AF3, AF4, F3, F4	10–20	✓
[69]	Neurofax μ EEG-9100	11	NA	NA	✓
[70]	BIOPAC (MP150)	8	NA	10–20	
[71]	g-MOBIlab (EEG)	3	NA	10–20	
[72]	NeuroScan	19	NA	10–20	
[73]	NA	3	Fz, Pz	10–20	✓
[74]	NeuroScan	13	Fp1, Fp2, F3, F4, Fz, C3, C4, Cz, P3, P4, Pz	10–20	✓
[75]	BIOPAC (EEG100A)	4	Cz, Pz, P3, P4	10–20	✓
[76]	NeuroScan	32	NA	E 10–20	
[77]	Emotiv EPOC system	14	NA	10–20	
[78]	BIOPAC (LXE1008C)	8	NA	10–20	
[79]	BIOPAC (EEG100A)	12	Cz, Pz, P3, P4, F3, F4, F7, F8, T3, T4, T5, T6	10–20	
[80]	NicoletOne Ambulatory EEG	16	NA	10–20	
[81]	NicoletOne Ambulatory EEG	16	NA	10–20	
[82]	Emotiv EPOC system	14	NA	10–20	
[83]	Emotiv EPOC system	14	NA	10–20	
[84]	Emotiv EPOC system	14	F3, F4 AF3, AF4, F3, F4	10–20	✓
[85]	Mindset	1	Fp1	10–20	
[86]	Mindset	1	Fp1	10–20	
[87]	Neurosky TGAM	4	Fp1, Fp2, TP9, TP10	10–20	✓
[88]	Emotiv EPOC system	14	O1, O2, P7, P8, T7, T8, FC5, FC6, F3, F4, F7, F8, AF3, AF4	10–20	✓
[89]	NeuroScan	30	NA	10–20	
[90]	BrainVision	10	F3, F4, P3, P4	10–20	✓
[91]	Mitsar-EEG-201	8	F3, F4, P3, P4, T3, T4, O1, O2	10–20	
[92]	ProComp EEG System	1	Pz	NA	
[93]	NeuroScan	62	NA	10–20	
[94]	Emotiv EPOC system	14	O1, O2, P7, P8, T7, T8, FC5, FC6, F3, F4, F7, F8, AF3, AF4	10–20	
[95]	QEEG-4 system	4	NA	NA	
[96]	NA	15	NA	10–20	

^1^ NAC ‘Number of Acquired Channels’. ^2^ CPA ‘Channels used for Power Analysis’ ground and reference excluded in counting. ^3^ EMS: ‘Electrode Montage System’; NA: ‘not available’; 10–20: International 10–20 system; E 10–20: Extended international 10–20 system. ^4^ CSF: ‘channels specified in formula’. When checkmark (✓) is present the information is reported in the original paper.

**Table 3:** Involvement indexes.

Index	Formula	Documents in Which the Index is Considered		
		Mental Strain	Sensory and Emotional Aspects	Movement
I_1_	$\frac{\beta }{\alpha }$	2, 5, 16, 18, 24, 27, 28, 35, 32, 46, 49, 52, 55, 56, 59, 67, 68, 71, 72, 74, 75, 76, 78, 86, 93	19, 42, 51, 54, 61, 84, 94	21, 58
I_2_	$\frac{\beta }{\alpha +\theta }$	5, 16, 18, 24, 27, 28, 31, 32, 35, 38, 41, 45, 47, 48, 49, 50, 52, 57, 63, 64, 67, 72, 74, 75, 76, 77, 78, 80, 81, 82, 83, 85, 87, 88, 89, 91, 93, 96	19, 26, 43	60
I_3_	$\frac{\beta }{\theta }$	18, 24, 27, 28, 29, 31, 34, 35, 46, 52, 53, 56, 62, 67, 69, 71, 72, 76, 79, 80, 89, 90, 93	19	60
I_4_	$\frac{\theta }{\alpha }$	18, 22, 24, 27, 30, 35, 36, 41, 44, 56, 69, 73, 74, 78, 80, 92		20, 23, 60
I_5_	$\frac{\theta }{\delta }$	33, 36, 56		
I_6_	$\frac{SMR}{\theta }$	34, 36, 37		
I_7_	$\frac{SMR}{\beta }$	36		
I_8_	$\frac{\alpha +\beta }{\delta }$	65		
I_9_	$\frac{\theta +\alpha }{\alpha +\beta }$	18, 24, 52, 67, 72, 80, 89, 93	19	
I_10_	$\frac{\theta }{\alpha +\beta }$	24, 46, 80		
I_11_	$\frac{\theta +\alpha }{\gamma }$	39		
I_12_	$\frac{\beta +\theta }{\alpha }$	25		
I_13_	$\frac{\delta +\theta }{\beta }$	24		
I_14_	$\frac{\delta +\theta +\alpha }{\beta }$	24		
I_15_	$\frac{\delta +\theta }{\alpha }$			60
I_16_	$\frac{\delta +\theta }{\alpha +\beta }$	18	40	60
I_17_	$\frac{\delta }{\alpha }$	56		
I_18_	$\frac{\delta }{\beta }$	18, 56	19	
I_19_	$\frac{\theta }{\gamma }$	46		
I_20_	$\frac{\alpha }{\gamma }$	46		
I_21_	$\frac{SMR+\beta }{\theta }$	70, 95		
I_22_	$\frac{\theta +\alpha }{\beta +\gamma }$	18	19	
I_23_	$\frac{\alpha +\beta }{\alpha +\theta }$	18	19	
I_24_	$\frac{\alpha }{\beta +\gamma }$	18	19	
I_25_	$\frac{\delta +\theta +\alpha }{\beta +\gamma }$	18	19	
I_26_	$\frac{\alpha }{\delta +\theta +\alpha }$	18		
I_27_	$\frac{\alpha }{\theta +\alpha +\beta }$	18		
I_28_	$\frac{\beta }{\theta +\gamma }$	18		
I_29_	$\frac{\beta +\gamma }{\delta }$	18		
I_30_	$\frac{\alpha +\beta }{\gamma }$	18		
I_31_	$\frac{\alpha +\gamma }{\theta +\delta }$	18		
I_32_	$\frac{\theta +\alpha }{\delta }$	18		
I_33_	$\frac{\theta +\beta }{\alpha +\gamma }$	18		
I_34_	$\frac{\beta +\gamma }{\delta +\theta }$	18		
I_35_	$\frac{\delta +\alpha }{\theta +\gamma }$	18		
I_36_	$\frac{\theta +\alpha }{\delta +\beta +\gamma }$	18		
I_37_	$\frac{\alpha +\beta }{\delta +\theta +\gamma }$	18		

## Data Availability

Not applicable since new data were created.

## Appendix A

### Appendix A.1. Pubmed

(eeg [Title/Abstract] OR electroencephalogra* [Title/Abstract]) AND ((“movement* control”[Title/Abstract]) OR (“movement* response”[Title/Abstract]) OR (“movement* task*”[Title/Abstract]) OR (“motor control”[Title/Abstract]) OR (“motor task*”[Title/Abstract]) OR (“motor response”[Title/Abstract]) OR (“language task*”[Title/Abstract]) OR (“language processing”[Title/Abstract]) OR (“language control”[Title/Abstract]) OR (“sensory control”[Title/Abstract]) OR (“sensory task*”[Title/Abstract]) OR (“sensory response”[Title/Abstract]) OR (emotion*[Title/Abstract]) OR (“mental state*”[Title/Abstract]) OR (“mental effort”[Title/Abstract]) OR (“mental fatigue”[Title/Abstract]) OR (“mental task*”[Title/Abstract]) OR (“mental load”[Title/Abstract]) OR (“mental function*”[Title/Abstract]) OR (“cognitive load”[Title/Abstract]) OR (“cognitive task*”[Title/Abstract]) OR (“cognitive effort”[Title/Abstract]) OR (“cognitive fatigue”[Title/Abstract]) OR (“cognitive function*”[Title/Abstract]) OR (stress[Title/Abstract]) OR (“working memory”[Title/Abstract]) OR (attention[Title/Abstract]) OR (vigilance[Title/Abstract])) AND ((wave*[Title/Abstract]) OR (“frequency band*”[Title/Abstract]) OR (oscillation*[Title/Abstract]) OR (rhythm*[Title/Abstract]) OR (“frequency content”[Title/Abstract]) OR (“frequency range”[Title/Abstract])) AND ((index* [Title/Abstract]) OR (indice*[Title/Abstract]) OR (characterization [Title/Abstract]) OR (marker* [Title/Abstract])) NOT (animal* [Title/Abstract]) NOT (stimul* [Title/Abstract]) NOT (disease* [Title/Abstract]) NOT (disorder* [Title/Abstract]) NOT (impairment [Title/Abstract]).

### Appendix A.2. Scopus

(TITLE-ABS(eeg) OR TITLE-ABS(electroencephalogra*)) AND (TITLE-ABS(“movement* control”) OR TITLE-ABS(“movement* response”) OR TITLE-ABS(“movement* task*”) OR TITLE-ABS(“motor control”) OR TITLE-ABS(“motor task*”) OR TITLE-ABS(“motor response”) OR TITLE-ABS(“language task*”) OR TITLE-ABS(“language processing”) OR TITLE-ABS(“language control”) OR TITLE-ABS(“sensory control”) OR TITLE-ABS(“sensory task*”) OR TITLE-ABS(“sensory response”) OR TITLE-ABS(emotion*) OR TITLE-ABS(“mental state*”) OR TITLE-ABS(“mental effort”) OR TITLE-ABS(“mental fatigue”) OR TITLE-ABS(“mental task*”) OR TITLE-ABS(“mental load”) OR TITLE-ABS(“mental function*”) OR TITLE-ABS(“cognitive load”) OR TITLE-ABS(“cognitive task*”) OR TITLE-ABS(“cognitive effort”) OR TITLE-ABS(“cognitive fatigue”) OR TITLE-ABS(“cognitive function*”) OR TITLE-ABS(stress) OR TITLE-ABS(“working memory”) OR TITLE-ABS(attention) OR TITLE-ABS(vigilance)) AND (TITLE-ABS(wave*) OR TITLE-ABS(“frequency band*”) OR TITLE-ABS(oscillation*) OR TITLE-ABS(rhythm*) OR TITLE-ABS(“frequency content”) OR TITLE-ABS(“frequency range”)) AND (TITLE-ABS(index*) OR TITLE-ABS(indice*) OR TITLE-ABS(characterization) OR TITLE-ABS( marker*)) AND NOT (TITLE-ABS(animal*)) AND NOT (TITLE-ABS(stimul*)) AND NOT (TITLE-ABS(disease*)) AND NOT (TITLE-ABS(disorder*)) AND NOT (TITLE-ABS(impairment)).

### Appendix A.3. Web of Science

(TI=(eeg) OR AB=(eeg) OR TI=(electroencephalogra*) OR AB=(electroencephalogra*)) AND (TI=(“movement* control”) OR AB=(“movement* control “) OR TI=(“movement* response”) OR AB=(“movement* response”) OR TI=(“movement* task*”) OR AB=(“movement* task*”) OR TI=(“motor control”) OR AB=(“motor control”) OR TI=(“motor task*”) OR AB=(“motor task*”) OR TI=(“motor response”) OR AB=(“motor response”) OR TI=(“language task*”) OR AB=(“language task*”) OR TI=(“language processing”) OR AB=(“language processing”) OR TI=(“language control”) OR AB=(“language control”) OR TI=(“sensory control”) OR AB=(“sensory control”) OR TI=(“sensory task*”) OR AB=(“sensory task*”) OR TI=(“sensory response”) OR AB=(“sensory response”) OR TI=(emotion*) OR AB=(emotion*) OR TI=(“mental state*”) OR AB=(“mental state*”) OR TI=(“mental effort”) OR AB=(“mental effort”) OR TI=(“mental fatigue”) OR AB=(“mental fatigue”) OR TI=(“mental task*”) OR AB=(“mental task*”) OR TI=(“mental load”) OR AB=(“mental load”) OR TI=(“mental function*”) OR AB=(“mental function*”) OR TI=(“cognitive load”) OR AB=(“cognitive load”) OR TI=(“cognitive task*”) OR AB=(“cognitive task*”) OR TI=(“cognitive effort”) OR AB=(“cognitive effort”) OR TI=(“cognitive fatigue”) OR AB=(“cognitive fatigue”) OR TI=(“cognitive function*”) OR AB=(“cognitive function*”) OR TI=(stress) OR AB=(stress) OR TI=(“working memory”) OR AB=(“working memory”) OR TI=(attention) OR AB=(attention) OR TI=(vigilance) OR AB=(vigilance)) AND (TI=(wave*) OR AB=(wave*) OR TI=(“frequency band*”) OR AB= (“frequency band”) OR TI=(oscillation*) OR AB=(oscillation*) OR TI=(rhythm*) OR AB=(rhythm*) OR TI=(“frequency content”) OR AB=(“frequency content”) OR TI=(“frequency range”) OR AB=(“frequency range”)) AND (TI=(index*) OR AB=(index*) OR TI=(indice*) OR AB=(indice*) OR TI=(characterization) OR AB=(characterization) OR TI=(marker*) OR AB=(marker*)) NOT (TI=(animal*) OR AB=(animal*) OR TI=(disorder*) OR AB=(disorder*) OR TI=(stimul*) OR AB=(stimul*) OR TI=(disease*) OR AB=(disease*) OR TI=(impairment) OR AB=(impairment)).

### Appendix A.4. IEEE Explore

(((((“Document Title”:eeg OR “Document Title”:electroecephalogra*) AND (“Document Title”:”movement control” OR “Document Title”:”movements control” OR “Document Title”:”movement response” OR “Document Title”:”movements response” OR “Document Title”:”movement task” OR “Document Title”:”movements task” OR “Document Title”:”movement tasks” OR “Document Title”:”movements tasks” OR “Document Title”:”motor control” OR “Document Title”:”motor task” OR “Document Title”:”motor tasks” OR “Document Title”:”motor response” OR “Document Title”:”language task” OR “Document Title”:”language tasks” OR “Document Title”:”language processing” OR “Document Title”:”language control” OR “Document Title”:”sensory control” OR “Document Title”:”sensory task” OR “Document Title”:”sensory tasks” OR “Document Title”:”sensory response” OR “Document Title”:emotion OR “Document Title”:emotions OR “Document Title”:”mental state” OR “Document Title”:”mental states” OR “Document Title”:”mental effort” OR “Document Title”:”mental fatigue” OR “Document Title”:”mental task” OR “Document Title”:”mental tasks” OR “Document Title”:”mental load” OR “Document Title”:”mental function” OR “Document Title”:”mental functions” OR “Document Title”:”cognitive load” OR “Document Title”:”cognitive task” OR “Document Title”:”cognitive tasks” OR “Document Title”:”cognitive effort” OR “Document Title”: “cognitive fatigue” OR “Document Title”:”cognitive function” OR “Document Title”:”cognitive functions” OR “Document Title”:stress OR “Document Title”:”working memory” OR “Document Title”:attention OR “Document Title”:vigilance) AND (“Document Title”:wave OR “Document Title”:waves OR “Document Title”:”frequency band” OR “Document Title”:”frequency bands” OR “Document Title”:oscillation OR “Document Title”:oscillations OR “Document Title”:rhythm OR “Document Title”:rhythms OR “Document Title”:”frequency content” OR “Document Title”:”frequency range”) AND (“Document Title”:index* OR “Document Title”:indice* OR “Document Title”:marker OR “Document Title”:markers OR “Document Title”:characterization)) OR ((“Abstract”:eeg OR “Abstract”:electroecephalogra*) AND (“Abstract”:”movement control” OR “Abstract”:”movements control” OR “Abstract”:”movement response” OR “Abstract”:”movements response” OR “Abstract”:”movement task” OR “Abstract”:”movements task” OR “Abstract”:”movement tasks” OR “Abstract”:”movements tasks” OR “Abstract”:”motor control” OR “Abstract”:”motor task” OR “Abstract”:”motor tasks” OR “Abstract”:”motor response” OR “Abstract”:”language task” OR “Abstract”:”language tasks” OR “Abstract”:”language processing” OR “Abstract”:”language control” OR “Abstract”:”sensory control” OR “Abstract”:”sensory task” OR “Abstract”:”sensory tasks” OR “Abstract”:”sensory response” OR “Abstract”:emotion OR “Abstract”:emotions OR “Abstract”:”mental state” OR “Abstract”:”mental states” OR “Abstract”:”mental effort” OR “Abstract”:”mental fatigue” OR “Abstract”:”mental task” OR “Abstract”:”mental tasks” OR “Abstract”:”mental load” OR “Abstract”:”mental function” OR “Abstract”:”mental functions” OR “Abstract”:”cognitive load” OR “Abstract”:”cognitive task” OR “Abstract”:”cognitive tasks” OR “Abstract”:”cognitive effort” OR “Abstract”: “cognitive fatigue” OR “Abstract”:”cognitive function” OR “Abstract”:”cognitive functions” OR “Abstract”:stress OR “Abstract”:”working memory” OR “Abstract”:attention OR “Abstract”:vigilance) AND (“Abstract”:wave OR “Abstract”:waves OR “Abstract”:”frequency band” OR “Abstract”:”frequency bands” OR “Abstract”:oscillation OR “Abstract”:oscillations OR “Abstract”:rhythm OR “Abstract”:rhythms OR “Abstract”:”frequency content” OR “Abstract”:”frequency range”) AND (“Abstract”:index* OR “Abstract”:indice* OR “Abstract”:marker OR “Abstract”:markers OR “Abstract”:characterization))) NOT (“Document Title”:animal OR “Abstract”:animal OR “Document Title”:animals OR “Abstract”:animals) NOT (“Document Title”:stimul* OR “Abstract”:stimul*) NOT (“Document Title”:disease OR “Abstract”:disease OR “Document Title”:diseases OR “Abstract”:diseases) NOT (“Document Title”:disorder OR “Document Title”:disorders OR “Abstract”:disorder OR “Abstract”:disorders) NOT (“Document Title”:impairment))).
